# A Comprehensive Review of Post-traumatic Injuries Among Formula 1 Drivers From 1950 to 2023: An Epidemiological Study

**DOI:** 10.5435/JAAOSGlobal-D-25-00055

**Published:** 2025-04-29

**Authors:** Johann P. Braithwaite, Shawn J. Geffken, Anthony Modica, Randy M. Cohn, Adam D. Bitterman

**Affiliations:** From the Northwell, New Hyde Park, NY (Dr. Braithwaite, Dr. Modica, Dr. Cohn, and Dr. Bitterman); the Department of Orthopaedic Surgery, Northwell Health Huntington Hospital, Huntington, NY (Dr. Braithwaite, Dr. Modica, Dr. Cohn, and Dr. Bitterman); the Zucker School of Medicine at Hofstra/Northwell, Hempstead, NY (Dr. Braithwaite, Dr. Modica, Dr. Cohn, and Dr. Bitterman); and the NYIT College of Osteopathic Medicine, Old Westbury, NY (Mr. Geffken).

## Abstract

**Purpose:**

To investigate the trends of injuries and mortalities throughout the 73-year history of Formula One (F1), and identify factors influencing driver injury and return to racing.

**Methods:**

Public online archives were searched to compile injury and driver characteristics of all F1 drivers between 1950 and 2023. The F1 drivers' Wikipedia articles were reviewed for injuries or mortalities from F1 events. The sources for each injury or mortality were reviewed and cross-referenced with additional sources to ensure accuracy. The injuries were further subdivided by anatomical location to analyze overall trends in incidence. Injury incidence and significance trends were determined via the Pearson correlation coefficient, and binomial logistic regressions were used to determine the relationships between driver characteristics and injuries.

**Results:**

The analysis included 865 F1 drivers. Overall, 264 total injuries and 43 deaths were reported in F1-related events. Across the analysis period, notable decreases were observed in total injuries (*R* = −0.48, *P* < 0.001), deaths (*R* = −0.56, *P* < 0.001), fractures (*R* = −0.42, *P* < 0.001), upper extremity injuries (*R* = −0.28, *P* = 0.007), lower extremity injuries (*R* = −0.29, *P* = 0.006), head injuries (*R* = −0.301 *P* = 0.003), torso injuries (*R* = −0.36, *P* < 0.001), internal injuries (*R* = −0.27, *P* = 0.01), and burns (*R* = −0.25, *P* = 0.017). Injury was more likely with more race entries (odds ratio [OR] = 1.01, CI = 1.004 to 1.013, *P* < 0.001) and less likely with increasing career racing points (OR = 0.998, CI = 0.996 to 0.999, *P* = 0.009). Drivers with lower extremity injuries were more likely to return to sport (OR = 2.89, CI = 1.36 to 6.16, *P* = 0.006) and less likely after experiencing internal (OR = 0.267, CI = 0.09 to 0.75, *P* = 0.013), head (OR = 0.485, confidence interval [CI] = 0.27 to 0.88, *P* = 0.017), and neck injuries (OR = 0.388, CI = 0.15 to 0.98, *P* = 0.046).

**Conclusion:**

The evolution of safety regulations in F1 appears to have successfully reduced total injuries, total deaths, and most injury classifications.

Formula One (F1) is the highest class of motorsport racing using open wheel, single-seater cars since its inception in 1950. The sport historically began with hazardous conditions and minimal safety regulations, such as open cockpits and unsafe racetracks—the lack of regulations led to frequent injuries and mortalities. In response, the governing body of F1, the Federation Internationale de l’Automobile, has continuously evolved safety regulations and technology to mitigate the risk to drivers and pit crew (Table 1).

**Table 1:** Formula One Safety Measures

Safety Measure	Year Implemented	Description
Helmet mandates	1952	Helmets now required during races
Hay bale barriers	1967	Hay bales used as collision barriers
Fire resistant suits	1975	Must withstand being heated to 600-800 degrees for 11 seconds
Survival cell	1981	Carbon fiber and Kevlar portion of the car surrounding where the driver is seated
Refueling ban	1984-1994, 2010+	Refueling no longer permitted during races
Safety car	1993	Car that limits race pace when track conditions are hazardous
Corner reworks	1994	Tracks adapted to neutralize dangerous corners
Pitlane speed limits	1994	Drivers limited to 80 km/h while driving in the pitlane
Headrests	1996	Modern headrest padding introduced
Wheel tethers	1999, 2011^a^	Tethers that prevent wheel detachment during accidents
Carbon fiber helmets	2001	Helmets made from carbon fiber introduced
Head and neck support device (HANS)	2003	System that anchors a drivers helmet to a carbon fiber collar, preventing hyperextension
In-ear driver accelerometers	2014	Tracks forces and driver head movements during accidents
Improved barriers	2015	Barriers updated after a driver's car was wedged under the top layer
Driver facing camera	2016	Allows for driver visualization during accidents
Halo	2018	Cockpit protection device that shields a drivers head from flying debris
Biometric gloves	2018	Gloves that track driver pulse and blood oxygen content
Fire protective gloves	2021	Gloves made with fire-resistant materials begin trials

a Wheel tethers were initially introduced in 1999, the number of tethers per wheel doubled in 2011.

F1 drivers constantly face the threat of injury during one of their many testing, practice, qualification, or race events. Studies have demonstrated that drivers are subject to immense force during acceleration, braking, cornering, and crashing at high speeds. They can encounter 5× gravitational (G) forces during races and forces up to 254× G during crashes.^1,2^ Wertman et al^3^ studied the incidence of upper extremity injuries in National Association for Stock Car Auto Racing drivers, citing upper extremity fractures as the most common injury, followed by neuropathies (carpal/cubital tunnel) from prolonged steering wheel vibrations and grasping. Similarly, Masmejean et al^4^ demonstrated several orthopaedic wrist injuries and neuropathies in F1 drivers during the 1998 season.

Despite our intuitive understanding of the dangers in motorsport, there is a paucity of available injury data or orthopaedic research. Research on motorsport injury is limited, with only five PubMed indexed studies in 2016.^3^ The Federation Internationale de l’Automobile has recently launched the World Accident Database, which will source data from racing accidents worldwide to expand research and understanding of racing injuries.^5^ Although a step in the right direction, it is unclear if the World Accident Database data will be available to the public, and the effects of its application will not be known for some time.

With the increase in popularity of the sport, one can expect more attention and financial resources to propel more performance and safety advancements. The authors sought to fill the existing gap in F1 research by providing an epidemiological analysis of orthopaedic injuries. The purpose of this study is (1) to report on a database of injuries in F1 from all available public data, (2) to evaluate the overall trends in injuries and mortalities in its 73-year history, and (3) to identify risk factors for injury based on driver characteristics. We hypothesize that there will be a notable decrease in injuries and mortalities secondary to evolving safety regulations over the many years of analysis.

## Methods

Eight hundred sixty-five drivers were identified through the driver database located on the F1 results website. The F1 drivers' Wikipedia articles were reviewed for driver accidents, injury information, and injury setting (F1 event versus non-F1 event). Driver characteristics and racing history were obtained from the F1 results website.^6^ The sources for each injury or mortality cited in the drivers' Wikipedia article were reviewed and cross-referenced with additional primary and secondary public online archives. The additional sources included F1 website archives, newspaper articles, interviews, autobiographies, biographies, and documentaries.^7,8^ Data collection included all the years between the inception of F1 (1950) and the end of the 2023 season. The following keywords were used to aid in the search: “accident,” “bone,” break,” “broken,” “crash,” “fracture,” “hurt,” “injury,” and “killed.” The data were collected by two researchers who independently and cross-referenced to limit inaccuracies.

The injuries sustained in an F1 event (test, practice, qualifying, grand prix race) were then identified. Injuries unrelated to an F1 event or without sufficient evidence were excluded. Driver characteristics were collected, such as race entries, fastest laps, points, race wins, podiums, and pole positions. Finally, the consequence of the injury (return to racing versus fatality/nonreturn) was collected.

Injuries were classified as (1) upper extremity; (2) lower extremity; (3) back and spine; (4) head; (5) neck; (6) torso; (7) pelvis/sacrum; (8) internal injuries; (9) burns; and (10) fractures (Table 2). Injuries sustained in a crash were recorded as present or absent in each designated classification. Multiple injuries sustained in a polytrauma crash were recorded as separate injuries and a single crash.

**Table 2:** Injury Classifications

Upper Extremity	Humeral head to distal phalanges of the hands
Lower extremity	Femoral head to distal phalanges of the toes
Back/spine	Thoracic and lumbar vertebra, back muscle, unspecified spinal injuries
Head	Skull, facial bones, brain
Neck	Cervical vertebrae, neck musculature
Torso	Musculoskeletal thoracic structures, ribs, clavicle, sternum
Pelvis/sacrum	Sacrum, ilium, ischium, coccyx, sacral-pelvic articulations
Internal	Organ, nonspecific internal injuries
Burns	Burns to any location
Fractures	All reported broken or fractured bones from any sub-classification

Statistical analysis was done using *jamovi*. Injury incidence and significance trends were determined through the Pearson correlation coefficient. Binomial logistic regressions were used to determine the relationships between driver characteristics and injury frequency, types of injuries, and rates of return to racing. Significance was defined as any *P* value of <0.05. Level IV evidence.

## Results

### Demographics

**Figure 1:**
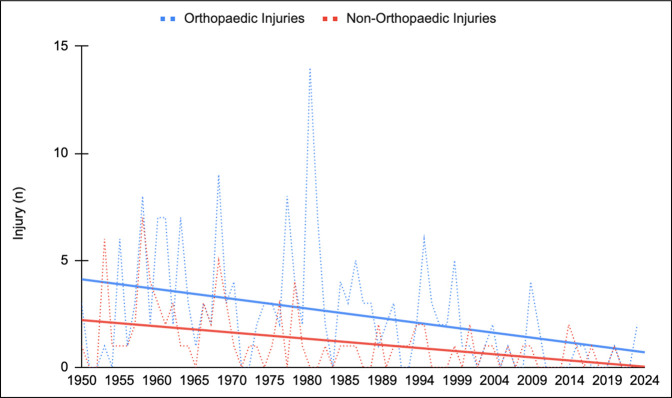
Graph showing trends in orthopaedic and nonorthopaedic injuries. Notable decreases were observed for both classifications.

The comprehensive evaluation identified 865 F1 drivers, which composed the study cohort. Overall, 264 driver injuries and 43 deaths were reported directly related to F1 events (testing, practice, qualifying, or Grand Prix races; Table 3). Orthopaedic injuries comprised 68.2% (180/264) of all injuries and decreased markedly over our study period (*R* = −0.37, *P* < 0.001). Non-orthopaedic injuries were less common (84/264, 31.8%), but similarly saw notable reductions (*R* = −0.44, *P* < 0.001; Figure 1).

**Table 3:** Injury Counts and Percentages

Factor or Variable	Injury, n (%)
Total injuries	264
Total deaths	42
Fractures	80 (32.5)
Upper extremity	20 (7.58)
Lower extremity	54 (20.5)
Back/spine	12 (4.55)
Head	37 (14.0)
Neck	10 (3.79)
Pelvis/sacrum	4 (1.52)
Torso	16 (6.06)
Internal	11 (4.17)
Burns	20 (7.58)

### Formula 1 Injuries

**Figure 2:**
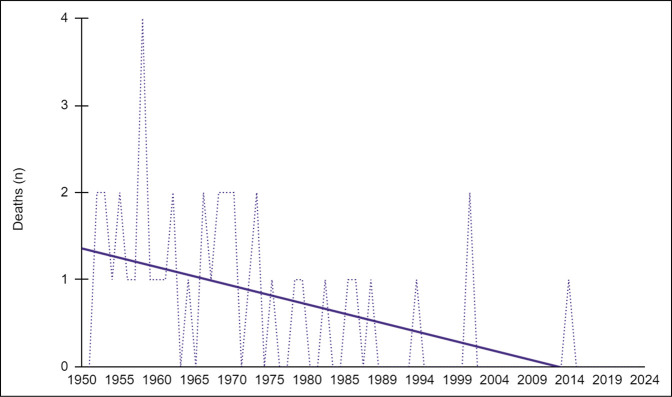
Graph showing trends in annual deaths over the study period. Notable decrease in annual deaths was observed over time.

**Figure 3:**
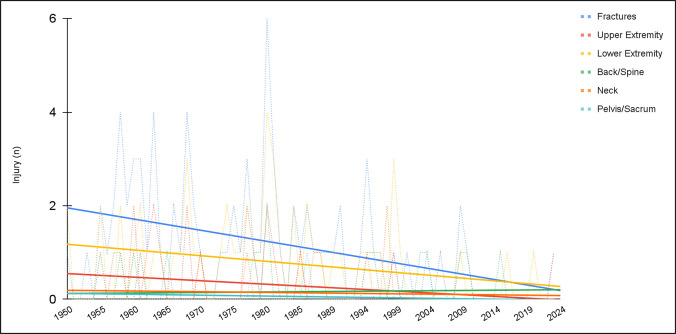
Graph showing trends in orthopaedic injuries over the study period. Notable decreases were observed for fractures, upper extremity injuries, and lower extremity injuries. No notable differences were observed for back/spine, neck, and pelvis/sacrum injuries.

We noted notable decreases in both total injuries (*R* = −0.48, *P* < 0.001) and deaths (*R* = −0.56, *P* < 0.001) over the analysis period (Table 4; Figure 2). When substratified, we found decreases in the incidence of fractures (*R* = −0.42, *P* < 0.001), upper extremity injuries (*R* = −0.28, *P* = 0.007), lower extremity injuries (*R* = −0.29, *P* = 0.006), head injuries (*R* = −0.301 *P* = 0.003), torso injuries (*R* = −0.36, *P* < 0.001), internal injuries (*R* = −0.27, *P* = 0.01), and burns (*R* = −0.25, *P* = 0.017). No notable trends were observed when evaluating the incidence of neck injuries (*R* = −0.09, *P* = 0.24), back/spine injuries (*R* = 0.06, *P* = 0.312), and pelvis/sacrum injuries (*R* = −0.18, *P* = 0.06; Figure 3).

**Table 4:** Trends in Driver Injuries Between 1950 and 2023

Factor or Variable	*R* ^2^	*R*	*P*
Total injuries	0.23	−0.48	<0.001
Deaths	0.32	−0.56	<0.001
Fractures	0.18	−0.42	<0.001
Upper extremity	0.08	−0.28	0.007
Lower extremity	0.08	−0.29	0.006
Back/spine	0.003	0.06	0.31
Head	0.10	−0.31	0.003
Neck	0.007	−0.09	0.24
Pelvis/sacrum	0.03	−0.18	0.06
Torso	0.13	−0.36	<0.001
Internal	0.07	−0.27	0.01
Burns	0.06	−0.25	0.02

Linear trends for annual injuries and deaths between 1950 and 2023.

### Risk Factors for Injuries

F1 drivers were more likely to experience an injury when they entered more races (OR = 1.01, CI = 1.004 to 1.013, *P* < 0.001; Table 5). As the number of points drivers earned during their careers increased, they became less likely to experience crash-related injuries (OR = 0.998, CI = 0.996 to 0.999, *P* = 0.009). We found no notable relationship between driver injuries and the number of race wins, pole positions, podiums, or fastest laps.

**Table 5:** Career Factors Predictive of Driver Injury

Factor or Variable	OR	Lower	Upper	*P*
Race entries	1.008	1.004	1.013	**<0.001**
Pole positions	1.052	0.945	1.171	0.385
Race wins	1.009	0.890	1.144	0.889
Podiums	1.065	0.988	1.147	0.101
Fastest laps	0.877	0.756	1.017	0.082
Points	0.998	0.996	0.999	**0.009**

Bolded numbers highlight statistically significant findings.

### Return to Racing

Injury classifications had varying effects on the driver's likelihood to return to racing. F1 drivers were less likely to return to racing after experiencing internal (OR = 0.267, CI = 0.09 to 0.75, *P* = 0.013), head (OR = 0.485, CI = 0.27 to 0.88, *P* = 0.017), and neck injuries (OR = 0.388, CI = 0.15 to 0.98, *P* = 0.046; Table 6). Drivers who experienced lower extremity injuries were more likely to return (OR = 2.89, CI = 1.36 to 6.16, *P* = 0.006). Fractures, upper extremity injuries, back/spine injuries, pelvis/sacrum injuries, torso injuries, and burns did not demonstrate any notable effect on the driver's likelihood of returning to racing.

**Table 6:** Likelihood to Return to Racing Post Injury

Factor or Variable	OR	Lower	Upper	*P*
Fractures	1.360	0.7216	2.564	0.342
Upper extremity	2.155	0.8517	5.453	0.105
Lower extremity	2.890	1.3564	6.158	**0.006**
Back/spine	2.365	0.8339	6.706	0.106
Head	0.485	0.2682	0.876	**0.017**
Neck	0.388	0.1535	0.982	**0.046**
Pelvis/sacrum	0.462	0.1018	2.098	0.317
Torso	0.848	0.3428	2.097	0.721
Internal	0.267	0.0943	0.754	**0.013**
Burns	0.749	0.3143	1.787	0.515

Bolded numbers highlight statistically significant findings.

## Discussion

We observed notable reductions in total annual injuries and deaths over the study period, which supported our hypothesis. When the injuries were subclassified, notable decreases were seen in fractures, upper extremity injuries, lower extremity injuries, head injuries, torso injuries, internal injuries, and burns. Injuries became more likely with increasing race entries and less likely as driver points increased. Drivers were less likely to return to motorsport after experiencing internal injuries, neck injuries, and head injuries and more likely to return after lower extremity injuries.

### Head and Neck

Head and neck injuries have historically been the leading cause of death in motorsport drivers before 2001.^9^ Our study demonstrated that the substantial effect of these injuries is not limited to driver mortality. F1 drivers were less likely to return to motorsport after experiencing either head (OR = 0.485, *P* = 0.017) or neck (OR = 0.388, *P* = 0.046) injuries. However, we observed a notable decrease in head injuries (*R* = −0.301 *P* = 0.003) over our analysis period, although no notable change was found in neck injuries. We anticipate that these changes are related to safety regulations, such as helmet mandates (1952), carbon fiber helmets (2001), head and neck support/HANS devices (2003), and Halo (2018; Table 1). In addition, in-ear accelerometers (2014) allow teams to track forces and driver head movement during crashes (Table 1). However, more research is needed to assess the direct effect of those safety regulations. Although severe head injuries may be declining, concussions are still prevalent in the motorsport community and may be rising.^10,11^ Adams et al^12^ conducted a survey study of motorsport drivers in 2020, where 31% reported they had concussions, demonstrating that although safety measures have improved, much work remains to be done.

### Back and Spine

Back and spine injuries did not have a notable change in incidence over the study period (*R* = 0.06, *P* = 0.312). Simulation models have determined that vertebral fractures could be reduced through cockpit modifications that permit greater forward pelvis movement.^13^ Modifications to seat position should also be carefully considered. Although they may reduce rates of acute spine injuries, if drivers are forced into different posturing positions, this may increase their risk of chronic low back and neck pain.^14^

### Orthopaedic Formula One Injuries

Notable decreases were observed in the annual incidence of fractures (*R* = −0.42, *P* < 0.001), upper extremity injuries (*R* = −0.28, *P* = 0.007), and lower extremity injuries (*R* = −0.29, *P* = 0.006). No notable trends were observed when evaluating the incidence of pelvis/sacrum injuries (*R* = −0.18, *P* = 0.06) (Table 4; Figure 3). We anticipate that these changes will be related to safety regulations such as the Survival cell (1981), safety cars (1993), corner reworks/pitlane speed limits (1994), and improved barriers (2005; Table 1). This study agreed with previous literature demonstrating a prevalence of upper extremity and lower extremity injuries in motorsport.^3,4,15^ However, this study demonstrated an overall positive effect of recent safety regulations.

### Risk Factors for Injury

We found that drivers who entered more races were more likely to experience injuries (OR = 1.02, *P* < 0.001). Interestingly, we found an inverse correlation between a driver's career success (racing points accumulated throughout the driver's career) and their likelihood of injury (OR = 0.998, *P* = 0.009; Table 5). This observation may be due to improved driving skills and experience. Outside of motorsports, novice drivers are at disproportionately higher risks of collisions.^6^ Conversely, drivers with more experience are better at adapting their driving performance in response to complicated driving environments.^7^ However, drivers who were not injured likely had more opportunities to accumulate more points. Finally, the number of annual races has increased from seven races in 1950 to 22 races in 2023.^6^ This likely allowed drivers to accumulate more points in the setting of improved safety regulations.

### Return to Racing

Our study found that drivers were more likely to return to racing after experiencing lower extremity injuries compared with other injury types (OR = 2.89, *P* = 0.006; Table 6). This may, in part, be due to advancements in lower extremity trauma rehabilitation, with early weight-bearing, pain management, and multimodal approaches to rehabilitation becoming increasingly popular.^16^ In addition, we anticipated that decreased lower extremity injury severity secondary to improved safety technology, such as the Survival Cell (1981), likely influenced this finding.

### Limitations

This study is not without limitations. We collected our data through various online public archives, and although the sources were cross-referenced with other online sources when applicable, there remains vulnerability to potential inaccuracies. Overall, a positive trend of safety regulations was found on orthopaedic injuries, nonorthopaedic injuries, and mortalities. However, we did not assess the injury effect of any specific change in safety equipment or regulation. The number of annual races has increased from seven races in 1950 to 22 races in 2023, which confounds annual comparisons across the study period. Additional research is warranted to determine safety regulations that had the most notable effect on injury incidence and mortality and to identify future areas for safety improvement. Finally, we acknowledge that the clinical applicability of this study is limited. However, with the recent surge in interest in F1, orthopaedic surgeons may begin to treat more motorsport injuries and a basic understanding of the incidence of these injuries is paramount.

## Conclusion

Overall, we observed statistically significant decreases in total injuries, deaths, fractures, upper extremity injuries, lower extremity injuries, head injuries, torso injuries, internal injuries, and burns following F1 crashes over our study period. More race entries increased the likelihood of injury, whereas driver points inversely correlated with the likelihood of injury. Finally, drivers with lower extremity injuries were more likely to return to racing than other injury patterns.

## References

[1] Jules Bianchi. Wikipedia. 2021. https://en.wikipedia.org/wiki/Jules_Bianchi. Accessed March 10, 2024.

[2] Küçükdurmaz F. Driver as a high level athlete. Sports Injuries. 2011;1:1121–1123.

[3] Wertman G Gaston RG Heisel W: Upper extremity injuries in NASCAR drivers and pit crew. Orthop J Sports Med 2016;4:232596711662942. 10.1177/2325967116629427 PMC4765815 26962541

[4] Masmejean EH Chavane H Chantegret A Issermann JJ Alnot JY: The wrist of the formula 1 driver. Br J Sports Med 1999;33:270-273. 10450483 10.1136/bjsm.33.4.270 PMC1756177

[5] WADB-World Accident Database: Federation Internationale de l'Automobile, 2015. https://www.fia.com/wadb-world-accident-database. Accessed March 10, 2024.

[6] Results: Formula 1®—The Official F1® Website. https://www.formula1.com/en/results.html. Accessed May 24, 2024.

[7] Historicracing.com: Historic Racing. https://www.historicracing.com/index.cfm. Accessed May 24, 2024.

[8] The “forgotten” Drivers of f1. The “Forgotten” Drivers of F1. https://www.f1forgottendrivers.com/. Accessed May 24, 2024.

[9] Kaul A Abbas A Smith G Manjila S Pace J Steinmetz M: A revolution in preventing fatal craniovertebral junction injuries: Lessons learned from the Head and Neck Support device in professional auto racing. J Neurosurg Spine 2016;25:756-761. 27401028 10.3171/2015.10.SPINE15337

[10] Deakin ND Hutchinson PJ: Concussion in motorsport: Incidence, awareness and future directions. Concussion 2017;2:CNC43. 30202584 10.2217/cnc-2017-0004 PMC6094153

[11] Finch CF Clapperton AJ McCrory P: Increasing incidence of hospitalisation for sport-related concussion in Victoria, Australia. Med J Aust 2013;198:427-430. 23641993 10.5694/mja12.11217

[12] Adams SA Turner AP Richards H Hutchinson PJ. Concussion in motorsport? Experience, knowledge, attitudes, and priorities of medical personnel and drivers. Clin J Sport Med. 2018;1:568–577. 10.1097/JSM.0000000000000647 30113965

[13] Katsuhara T Takahira Y Hayashi S Kitagawa Y Yasuki T. Analysis of driver kinematics and lower thoracic spine injury in World endurance championship race cars during frontal impacts. SAE Int J Transport Saf. 2017;5:120-132.

[14] Fernando F Biffi A Borra F De Carli F Monetti G Sirico F. Formula 1 World championship. Epidemiology of Injuries in Sports. Princeton, NJ: Springer; 2022:95–106.

[15] Stelzenbach C Valderrabano V. Motorsports. Foot Ankle Sports Orthopaedics. Princeton, NJ: Springer; 2016:505–508.

[16] Hoyt BW Pavey GJ Pasquina PF Potter BK: Rehabilitation of lower extremity trauma: A review of principles and military perspective on future directions. Curr Trauma Rep 2015;1:50-60.

